# Antidepressant-induced acute colonic (pseudo) obstruction (Ogilvie syndrome)

**DOI:** 10.4103/0019-5545.46080

**Published:** 2005

**Authors:** V.A.P. Ghorpade

**Affiliations:** * Professor and Head

**Keywords:** Ogilvie syndrome, intestinal pseudo-obstruction, antidepressants

## Abstract

Patients on antidepressant drugs commonly complain of dryness of the mouth, tremors, blurring of vision and constipation, which are attributed to the anticholinergic action of the drugs. We report two cases of gastrointestinal complications (pseudo-intestinal obstruction), which are considered rare according to a review of the literature. This condition is also known as Ogilvie syndrome.

## INTRODUCTION

The common occurrence of depressive disorders has led to the extensive use of antidepressant drugs by various specialists. Most clinicians are aware of the anticholinergic side-effects, such as blurring of vision, dryness of the mouth, constipation and retention of urine. Several studies report that acute abdomen due to the use of antidepressants is rare.^1^–^4^ Sood and Kumar reported a case of imipramine-induced colonic pseudo-obstruction as Ogilvie syndrome,^1^ though originally this name was given to intestinal obstruction with caecal perforation by Mc Mahon.^4^ Later, Ross *et al*., and Gupta and Narang reported similar cases with the use of imipramine hydrochloride.^2^,^3^ We report two cases to support the view that intestinal obstruction can be a complication of the use of antidepressant drugs.

## CASE 1

A 60-year-old woman was on treatment for recurrent depressive disorder for the past 10 years with fluoxetine 80 mg/day and amitriptyline 150 mg/day. She was brought for psychiatric consultation with complaints of not talking, tremulousness, constipation and inability to pass urine for the past 4 days.

Physical examination revealed mild tachycardia (112 beats per minute), a tense abdomen and distended bladder.

She was admitted to the hospital with a provisional diagnosis of depressive stupor with anticholinergic side-effects following the use of antidepressant drugs.

Her bladder was catheterized, the antidepressants were stopped and zopiclone hydrochloride 5 mg was given at bedtime.

On day 2, she developed signs and symptoms of intestinal obstruction and on day 3, the bowel sounds were absent.

An erect plain radiograph of the abdomen confirmed the diagnosis of distal bowel obstruction without any evidence of perforation.

With conservative management, her condition improved on day 4 and she was discharged on day 8.

## CASE 2

A 65-year-old male was on psychiatric treatment for obsessive-compulsive disorder for the past 8 years with clomipramine 50 mg/day and nitrazepam 10 mg/per day. Over the previous 3 months, his food intake had gradually decreased and he developed constipation. Over the past 1 week, he developed abdominal distension with pain. The consulting surgeon made a preoperative diagnosis of subacute large bowel obstruction due to a suspected carcinomatous lesion. The antidepressant was stopped and the patient was investigated. Plain radiographs of the abdomen revealed signs of obstruction with multiple faecoliths and no evidence of intestinal perforation. Barium enema revealed obstruction at the rectosigmoid junction and a sigmoid volvulus.

An exploratory laparotomy was done to rule out bowel cancer and a final diagnosis of atonic pelvic colon was made. The patient recovered completely.

## DISCUSSION

Acute colonic pseudo-obstruction or Ogilvie syndrome is characterized by massive dilatation of the colon. It can occur due to various medical and surgical conditions, and as a side-effect of antidepressants.^1^ The literature reveals 5 reports of such cases, predominantly in the elderly.^1^,^3^ Only 2 of these 5 cases were surgical emergencies.^2^ The cases reported here are similar to those described in the literature except that in 1 case surgical exploration was done to rule out bowel cancer. Recognition of the condition at an early stage and awareness of such complications enabled us to manage the first case conservatively, whereas preoccupation with the diagnosis of probable malignancy led to management with surgical exploration in the second case. A good rapport is required between the treating doctor and the psychiatrist. Such drug-induced complications will possibly become rare due to the introduction of specific serotonin reuptake inhibitors (SSRIs), which do not have anticholinergic side-effects. It is important to educate the elderly as well as patients in other age groups about the importance of diet and fluid intake to overcome constipation. The patients presented here were unlikely to have had discontinuation syndrome, as symptoms were present even before the drugs were discontinued. The discontinuation syndrome can occur due to abrupt stoppage of antidepressant drugs. The discontinuation syndrome can involve any system of the body, is usually transient in nature, emerges 24–48 hours after discontinuation, and lasts for 7–14 days.^5^ The two patients whose reports are discussed here also developed the above-mentioned complications within 24 hours of discontinuation of the antidepressant drugs. Thus, slow tapering of the dosage of this group of drugs is recommended.
